# Vickers Hardness and Shrinkage Stress Evaluation of Low and High Viscosity Bulk-Fill Resin Composite

**DOI:** 10.3390/polym12071477

**Published:** 2020-06-30

**Authors:** Allegra Comba, Nicola Scotti, Tatjana Maravić, Annalisa Mazzoni, Massimo Carossa, Lorenzo Breschi, Milena Cadenaro

**Affiliations:** 1 Department of Biomedical and Neuromotor Sciences, DIBINEM, University of Bologna, Alma Mater Studiorum, Via San Vitale 59, 40125 Bologna, Italy; alle_comba@yahoo.it (A.C.); tatjana.maravic2@unibo.it (T.M.); annalisa.mazzoni@unibo.it (A.M.); lorenzo.breschi@unibo.it (L.B.); 2 Department of Surgical Science, CIR Dental School, University of Turin, Via Nizza 230, 10126 Turin, Italy; massimo.carossa@unito.it; 3 Department of Medical Sciences, University of Trieste, Strada di Fiume 447, 34149 Trieste, Italy; mcadenaro@units.it; 4 Institute for Maternal and Child Health—IRCCS “Burlo Garofolo”, Via dell’Istria 65/1, 34137 Trieste, Italy

**Keywords:** bulk-fill composite, microhardness, shrinkage stress, composite resins

## Abstract

The aim of this in vitro study was to evaluate the hardness and shrinkage stress (SS) of six bulk-fill resin composites. To evaluate microhardness (MH), ten 6 mm specimens were prepared using a metal mold for each selected bulk-fill resin composite and irradiated from the top side for 40 s using an LED light. After 24 h of storage, Vickers MH was evaluated on the upper, lower and lateral sides of the specimens. SS evaluation was then performed with a universal machine, which evaluated the contraction force generated by a bulk-fill composite specimen placed between two metal cylinders during and after light curing. The results were evaluated with a one-way ANOVA test with a post-hoc Bonferroni test and linear regression analysis (*p* < 0.05). All materials showed a significant MH decrease between the top and bottom surfaces. However, the bulk-fill materials tested performed differently when considering lateral depth progression. ANOVA tests for SS evaluation showed that both SDR and Venus Bulk Fill had significantly lower stress during irradiation than other tested materials. Further, MH decrease became significantly lower from the top surface at different depths in each tested group. Among the different resins, Venus Bulk Fill and SDR showed not only inferior hardness, but also a significant reduction in SS.

## 1. Introduction

For most resin-based composite materials, incremental layering techniques have been accepted and are widely used, especially in high C-factor cavities [1,2]. Proper light curing is performed when a sufficient energy density is delivered to every resin composite layer, which cannot be greater than 2 mm for the material to be completely irradiated and, thus, cured [3]. Furthermore, it is known from the literature that resin irradiation induces a volumetric contraction of the material, which results in shrinkage stress (SS) at the adhesive interface [4,5]. SS can cause deflection of the cusps, enamel and dentinal cracks, post-operative sensitivity, inflammation of the pulp, and the detachment of the adhesive interface [5,6]. These events, in time, can lead to marginal infiltration and secondary caries, and thus, the failure of the resin composite restoration [7]. 

Recently, with the attempt to overcome some limitations of these materials, a new type of light-curing resin composites has been introduced, the so-called bulk-fill resin composites. These materials are characterized by an increased maximum increment thickness. According to manufacturers’ claims, they could be placed in layers of up to 4mm thick without compromising the polymerization and the degree of conversion [8,9], resulting in a need for fewer increments. To date, there have been few randomized clinical studies that evaluated the in vivo behavior of these materials: Van Dijken and Pallesen reported a comparable annual failure rate between bulk-fill resin composite (class 1: 1.1%; class 2: 1.4%) and conventional resin composite (class I: 1.3%; class 2: 2.1%) after 5 y of clinical function [10].

On the other hand, several in vitro studies focused on bulk-fill composites, confirming that the micro-mechanical properties and degree of conversion are satisfactory in layers of 4 mm polymerized for 20 s [9,11], supporting the claim that they can be cured in large increments. 

Manufacturers also claim that the SS of these new resin composites is even lower than that found both in flowable and in non-flowable composites; a recent study showed that minor SS exerted by bulk-fill flowable composites translates into a lower cuspal deflection compared to traditional composites placed with an oblique layering technique [12]. However, because of the lower mechanical properties [13] (hardness and modulus of elasticity are closely related to the amount of filler), the use of low-viscosity bulk-fill resin composite is not recommended in situations where high mechanical stress is present, such as in direct contact with occlusal loads. Previous findings [14] showed that the Young modulus, Vickers hardness, and indentation modulus classified some bulk-fill materials (SureFil SDR, Venus Bulk Fill and Filtek Bulk Fill) between hybrid and flowable composites. For this reason, bulk-fill composites with increased viscosity were more recently produced to overcome the mechanical limitations and increase clinical indications. 

Indeed, the classification of bulk-fill materials into low and high viscosity reflects their mechanical properties and determines the clinical procedure: restorations with low viscosity materials (SureFil SDR, Venus Bulk Fill, X-tra Base, Filtek Bulk Fill) must be finalized by placing a layer of conventional resin composite above them, while high-viscosity bulk-fill composites (Tetric EvoCeram Bulk Fill, SonicFill) do not need this final layer [14].

If bulk-fill composites are to provide a true clinical advantage, then they require high depth of cure while simultaneously demonstrating a decrease in internal stress, and a subsequent decreased incidence of internal gap formation [15]. Nevertheless, a recent study showed that bulk-fill materials, both flowable and non-flowable, resulted in a similar proportion of gap-free marginal interface when compared to a conventional composite [16]. 

The purpose of this in vitro study was to evaluate the hardness and SS of six bulk-fill resin composites. The null hypothesis was that (1) the decrease in hardness is not directly related to the increment thickness, and that (2) SS is not comparable among the various composites tested.

## 2. Materials and Methods

For this in vitro study, six bulk-fill resin composites available on the dental market were selected (Table 1). 

### 2.1. Hardness Evaluation

**Figure 1:**
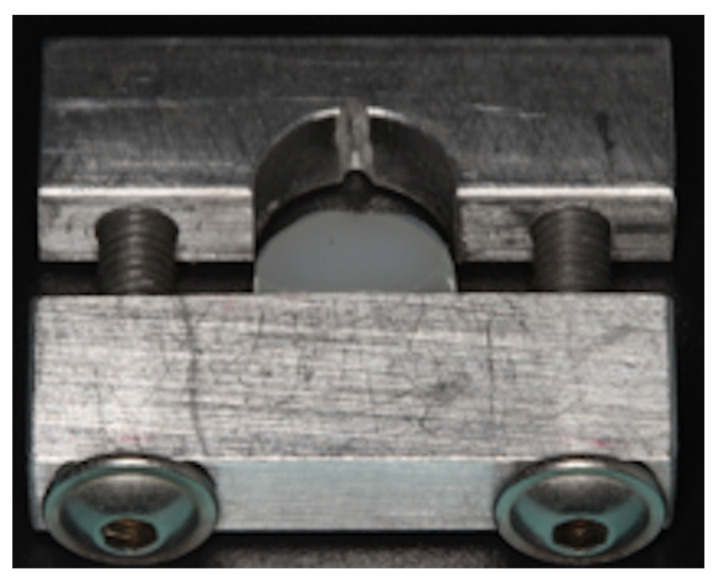
Semicircular metal molds with a diameter of 10 mm and a depth of 6 mm used in this study.

Ten specimens for each selected bulk-fill resin composite were prepared for a total of 60 specimens. Composites were placed with a bulk-fill technique inside semicircular custom-made stainless steel molds with a diameter of 10 mm and a depth of 6 mm (Figure 1).

The bottom surface of the mold was in contact with a glass plate, which simulated the pulpal floor of an ideal first-class cavity. The tip of the curing light (10 mm diameter) was placed in contact with the upper surface of the specimen, which was coated with a transparent Mylar strip with the aim of preventing the oxygen inhibition layer. Irradiation was performed for 40 s with a poly-wave LED light (Bluphase Style, IvoclarVivadent, Shaan, Lichtenstein), which was placed on the top of the samples with 2 different standardized orientations (20 sec each) to allow uniform irradiation with either blue or violet emitters. The irradiance of the curing unit was 1200 mW/cm^2^ as measured using a commercial dental radiometer (100 Optilux radiometer; SDS Kerr, Danbury, CT, USA). After 24 h of storage, the mold was opened to expose the lateral surface of the specimen, which was then polished with ascending 600–1000–2400 grit SiC paper for 3 min to remove from the top, bottom, and flat lateral surfaces the resin-rich layer formed against the matrix. Specimens were then ultrasonically cleaned in distilled water for 3 min and then stored in a dark container in air at 37 °C for 24 h.

**Figure 2:**
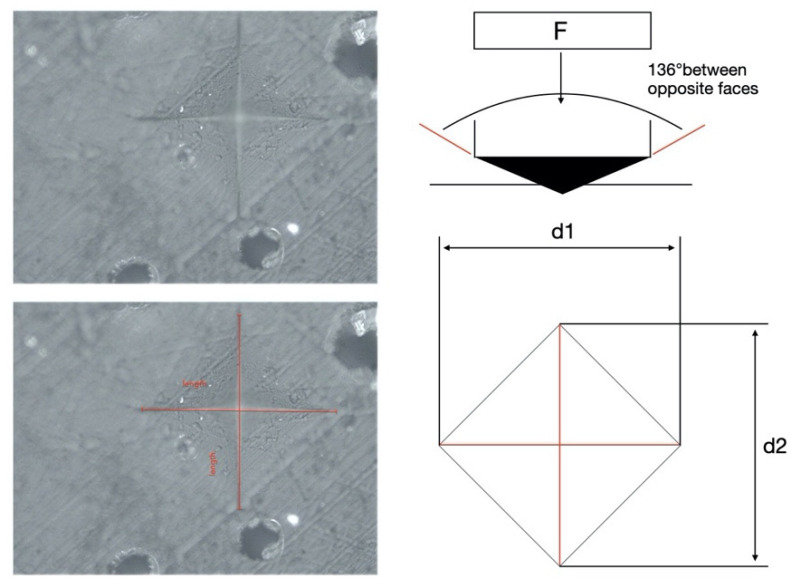
Vickers microhardness test.

The microhardness (MH) of each specimen was measured on the top (in contact with curing tip), bottom, and lateral surfaces using a Vickers indenter (Leica Microsystems S.p.A., Milano, Italy) at 100gf of load and 15 s dwell time. The mean MH value for each surface was calculated. For each specimen, 6 measurements were performed on the top and the bottom surfaces and 18 measurements were performed on the lateral surface, three for each millimeter, starting from the surface in contact with the curing light tip. The size of the indentation left by the tip was calculated with the aid of a microscope; the Vickers number (VHN) was calculated according to the following formula:

VHN = 1.854(F/D^2^)

where F is the applied load (measured in kilograms-force) and D^2^ is the area of the indentation (measured in square millimeters) as shown in Figure 2.

### 2.2. Shrinkage Stress Evaluation

SS evaluation was performed with a universal machine (Sun 500, Galdabini, Cardano al Campo, Italy) which takes into consideration the contraction force generated by a bulk-fill resin composite specimen placed between two metal cylinders during and after light curing. The evaluation setup was comprised of two stainless steel cylinders used as bonding substrates—with a 2 mm diameter and 25 mm height—which were attached to the upper and lower clamps of the universal machine. Prior to each measurement, the 2 mm surface of the stainless steel cylinder was treated in order to improve the retention of the testing machine. First, the attachments were sanded with 180-grit sandpaper and air-abraded using a silica-containing abrasive (Cojet,3M ESPE, St. Paul, MN, USA). A layer of hydrophobic unfilled resin (Optibond FL, Kerr, Danbury, CT, USA) was applied on the sandblasted surface and polymerized for 20 s with an LED curing unit (Bluephase Style, IvoclarVivadent, Shaan, Lichtenstein) before resin composite application to ensure appropriate bonding to the stress analyzer. 

The distance between the two cylinders was set at 2 mm (diameter 2 mm, height 2 mm; C-factor = 0.5). A Mylar film was placed around the rods and filled with the composite. An extensometer (model 2630-101, Instron, Norwood, MA, USA) was attached to the cylinders to provide an electronic feedback loop in the system to maintain the specimen at a constant height during the test. Any estimated movement between the cylinders of the extensometer of the bulk-fill resin composite shrinkage was immediately compensated for by controlled movement of the crosshead in the opposite direction (within 0.1 μm).

A defined quantity of bulk-fill resin composite (20 mg) for each tested material was placed in the mold in bulk technique and polymerized for 40 s. The curing light was always placed laterally to the resin composite specimen. The contraction force (N) generated during polymerization to maintain a constant specimen height in opposition to the force exerted by resin composite shrinkage was continuously recorded for 5 min after irradiation. Each experiment was conducted at room temperature (23–24 °C) and repeated ten times for each material (N = 10). SS (MPa) was calculated at 5 min as the force value (N) per area unit (force value/bonded surface area). SS, expressed in MPa, was calculated using the formula:

Shrinkage Stress (MPa) = Force (N)/Area (mm^2^)

### 2.3. Statistical Analysis

MH and SS differences among the tested bulk-fill composites were compared using a one-way ANOVA test with post-hoc Bonferroni correction. Furthermore, a linear regression was performed to obtain the depth at 80% of max MH of each material. Pearson correlation analysis was performed to calculate the correlation between stress values and MH. The significance level was set at 95% (*p* < 0.05). All statistical analyses were performed using Stata software package 12.0 (StataCorp, College Station, TX, USA). 

## 3. Results

### 3.1. Microhardness

Mean values and standard deviations of MH registered with the tested materials for the top, lateral (1 mm to 6 mm), and bottom surfaces are listed in Table 2. Different lower-case superscript letters (a, b) indicate differences within the same column.

In Table 3, the depth of cure, which was calculated as 80% of max VHN, is displayed in VHN and millimeters. 

**Figure 3:**
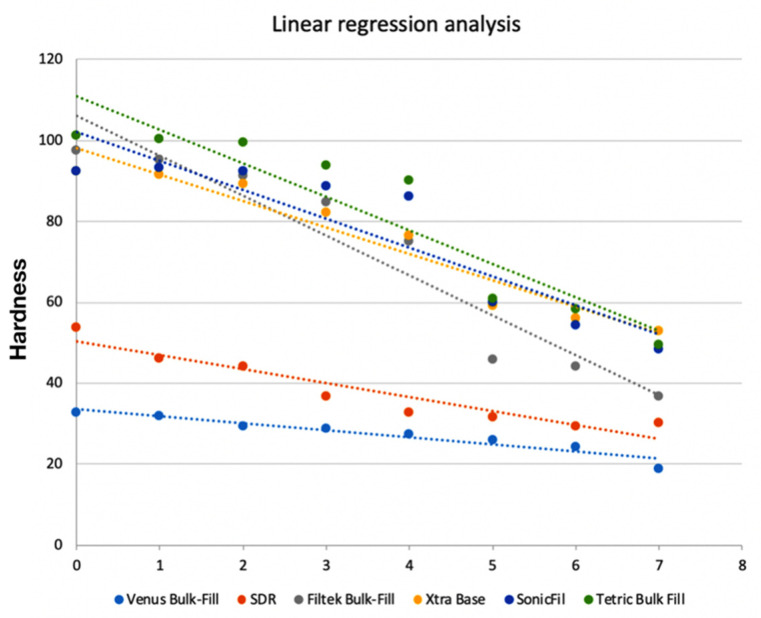
Linear regression of lateral MH progression of tested bulk-fill materials.

Figure 3 shows the linear regression of the lateral MH in relation to the depth of each group. 

Statistical analysis of variance showed that all materials had a significant MH decrease between the top and bottom surfaces (*p* < 0.001). However, the bulk-fill materials tested performed differently when considering lateral depth progression. Using top-surface MH values as the reference point, regression analysis showed that SDR had a significant difference at 2 mm depth; X-tra Base and Filtek Bulk Fill showed a significant difference at 3 mm depth; TetricBulk at 4mm; SonicFill at 4 mm; and Venus Bulk showed comparable MH values between the top and lateral surfaces at up to 5mm depth.

### 3.2. Shrinkage Stress

Mean SS (±SD), expressed in MPa, and the time to reach the max SS, expressed in seconds, of the tested materials are shown in Table 4.

**Figure 4:**
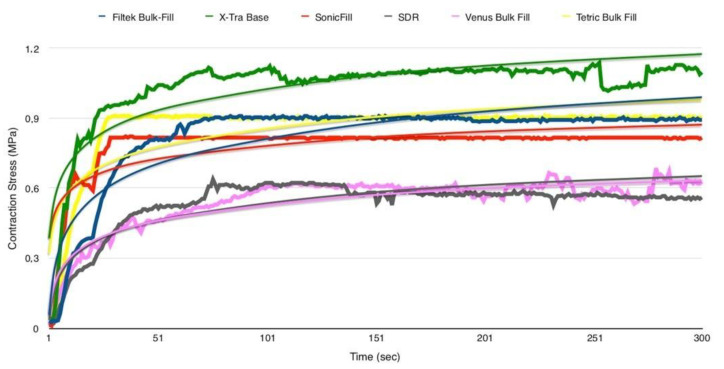
Plots of the SS (MPa) versus time (s) of the tested bulk-fill composites.

Representative SS versus time curves are shown in Figure 4.

**Figure 5:**
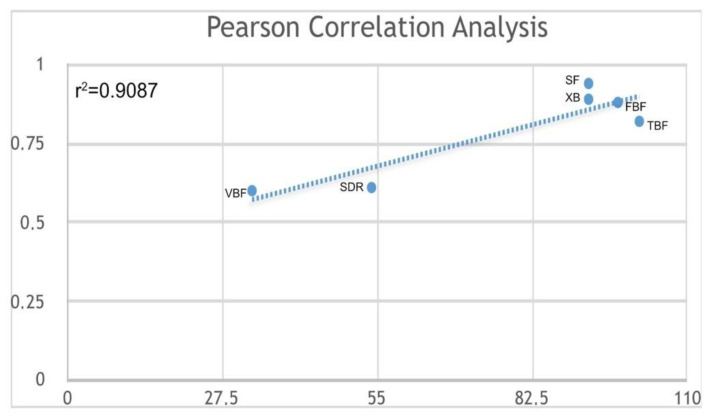
Pearson correlation of max. MH versus SS (MPa) for bulk-fill composites (Venus Bulk Fill (VB); SDR; Xtra-Base (XB); SonicFill (SF); Filtek Bulk Fill (FBF); Tetric Bulk Fill (TBF)).

The statistical analysis of variance for SS evaluation showed that both SDR and Venus Bulk-Fill presented significantly lower stress during irradiation than the other tested materials (*p* = 0.001). A significant direct correlation (*r* = 0.90, df=4, *p* < 0.05) was found between the stress values and microhardness of the tested materials (Figure 5).

## 4. Discussion

The results of the present study lead us to reject the first null hypothesis, since MH was significantly reduced with depth along the lateral surface of the materials tested. Thus, in all cases a significant decrease in MH values between the top and the bottom surfaces was found. Moreover, lateral surface MH analysis and final depth of cure showed different behaviors among the materials tested. 

Hardness is a mechanical property that indicates the resistance of a material to indentation or penetration, which is influenced by the filler characteristics (size, weight, volume) and the chemical composition of the resin [17]. A strong relationship between the amount of filler and the mechanical properties, such as hardness and elastic modulus, has been reported [18,19]. The resin composite hardness is usually measured using the Vickers [8] or Knoop [20,21] methods. These techniques provide an indentation using a diamond tip, which exerts a pre-established force for a certain time. Hardness is then obtained by dividing the applied load by the area of indentation, examined through a microscope, and multiplied by a given coefficient. This method has the advantage of being relatively simple, reproducible, and non-destructive [22,23]. Moreover, advances in instrumentation have made indentation a useful research tool for many different systems across size scales (macro to nano) and numerous scientific disciplines. For these reasons, the hardness of the materials tested in this study was evaluated by Vickers testing with an applied force of 100 g for 15 s [24].

In the present in vitro study, the bulk-fill materials tested showed different MH values when considering the top surface of the specimens. These findings are in accordance with other studies that compare various bulk-fill and traditional resins [25,26]. Differences in top-surface MH among bulk-fill materials can be mainly attributed to the great variety of filler size and content [27]. In this study, Venus Bulk Fill and SDR were among the materials with lower MH values. These findings are in accordance with other studies showing the reduced filler percentages of these two materials [28,29]. However, different materials, such as Filtek Bulk Fill, showed a reduced percentage in filler content and volume, but higher top-surface MH. Indeed, MH can also be attributed to other factors not related to filler content, but strictly associated with matrix composition and shrinkage behaviors [30].

Since mechanical properties are directly proportional to the number of double bonds involved in the polymerization reaction and, therefore, the resin composite degree of conversion [31], MH could be effectively considered an indirect method of assessing the polymerization quality of composites [30,32]. As stated by Leprince et al. [33], the MH could be considered an “indirect approximation” of the depth of cure. Indeed, the degree of conversion evaluation through MH gives results comparable to those obtained with a direct method, such as Fourier infrared spectroscopy (FTIR micro-MIR) [31,33]. 

The depth of cure was defined in the literature by Musanje and Darvell [34] as the depth at which the hardness is equal to 80% of the surface hardness. In resin composites it depends on several factors: the size and type of filler [33], color and translucency [35,36], material thickness [35], curing light intensity [35,37,38], irradiation time and program [39], and the distance between the resin composite surface and the curing light tip [35]. Moreover, the monomer composition and photoinitiator concentration [40] are also able to influence the depth of cure. This is in accordance with the Lambert–Beer law [41], which states that light energy, incident to the surface of a material, is affected, in an attempt to pass through it, by an attenuation coefficient, which is proportional to the physical characteristics of the material itself [42]. The method frequently employed to evaluate the depth of cure is ISO 4049 [31]: the resin composite to be tested is inserted into a mold and cured, and then is pulled out from the mold and the uncured resin is scraped off with a spatula. Finally, the height of the specimen is measured and the residual height divided by 2; the value obtained indicates the depth of cure and defines the maximum increase that can be achieved with the resin composite tested. In a study conducted by Flury et al. [31], ISO 4049 was compared to the Vickers MH test to determine bulk-fill resin composite depth of cure. Results showed that ISO 4049 tended to overestimate depth of cure when compared to Vickers MH, which gave the depth at which at least 80% of maximum hardness was obtained. More recently, even with bulk-fill materials, several papers have evaluated the depth of cure as the 80% hardness drop-off. [43,44]. 

In the present study, hardness was measured on the lateral surface of the specimen 24 h after irradiation with the same laboratory [29] and storage conditions not to alter the results of the test [45]. All the materials tested showed a significant decrease from the top surface towards the bottom. X-traBase, Filtek Bulk Fill, SonicFill, and Tetric Bulk Fill showed significantly different hardness at about 4 mm from the top surface, confirming the results of previous studies in the literature that assessed the depth of cure [46] and, thus, supporting manufacturer suggestions [30,47]. These results generally confirm manufacturers’ specifications, and a previous report [33] stated that bulk-fill materials could be placed in 4 mm thick layers instead of using the traditional incremental placement technique, without negatively affecting polymerization shrinkage, cavity adaptation, or degree of conversion (DC). However, as shown in Figure 3, not all materials had comparable trend curves. SonicFill, Xtra-Base, TetricBulk Fill and Filtek Bulk Fill behaved in a similar manner, showing a vertical decrease in hardness at 4 mm. Venus Bulk Fill, on the contrary, showed a more linear trend, with a significant difference from top surface hardness only at 4.92 mm. The behavior of this material can be related to its composition and consequently more homogeneous stress distribution [25]. Considering the results of this study, SDR was the only material that showed a significant decrease in MH at a depth inferior to that suggested by the manufacturer. The significant decrease for this material was evident at 2.15 mm depth and the same results were obtained when depth of cure was calculated. This finding was not in agreement with previous reports that confirmed the depth of cure claimed by SDR’s manufacturer [30,48]. 

When considering shrinkage stress, statistically significant differences were found between the tested materials, and accordingly, the second null hypothesis was accepted. On the other hand, there was no statistically significant difference between SDR and Venus Bulk Fill, which exhibited the lowest SS during irradiation. It is worth mentioning that in the setting of this in vitro study, we provided a specific environment that allowed for comparisons of the behavior of the tested materials under standardized conditions, but the results may vary under different testing conditions, especially with an increased C-factor. It can be further affirmed that the stress development of tested bulk-fill materials was mainly based on matrix composition and structure and filler content [49], as well as microhardness. In fact, a significant correlation was found between the SS and MH data.

In addition to the hardness of a material, the amount of filler can also influence the elastic modulus of a resin composite [50]. Several studies have reported that the elastic modulus increases exponentially with increasing filler concentration [51,52]. Consequently, the volumetric shrinkage is strictly dependent on the filler amount [53,54]. The amount of filler reduces the volume occupied by the resin matrix and, therefore, the number of methacrylate groups, leading to a lower volumetric shrinkage. Previous findings [55,56] showed a linear relationship between SS and elastic modulus, thus associating SS and filler concentration. The results of this study, however, showed an inverse relationship between SS and filler content. This discrepancy is due to the different evaluation methods used to assess shrinkage stress. As shown by Marchesi et al. [57], high-compliance testing methods are related to low values of shrinkage stress, while low-compliance testing methods could lead to an overestimation of shrinkage stress. The materials used in the present study that showed a SS significantly lower than the other materials tested included SDR and Venus Bulk Fill. The low SS of these materials is attributable to their low elastic modulus, due to a reduced amount of filler in their volume, which increases the flexibility of the material and, therefore, the ability to internally absorb stresses [58]. This viscoelastic behavior is typical of flowable materials, and is confirmed by the time required to achieve the maximum stress rate of the tested bulk-fill composites. The present results clearly show that low-viscosity materials reached the highest stress rate around 40 s after light exposure ended. Braga and Ferracane [5] explained that SS is an extremely complex multifactorial phenomenon. It is both related to volumetric shrinkage during the polymerization of the resin composite material that is bonded to cavity walls, and to its viscoelastic behavior (the ability to flow internally during polymerization), either of which could affect the elastic modulus. Composites with high filler content provide low shrinkage but higher stiffness than materials with lower filler concentration [5]. On the other hand, the increase in the degree of conversion of the resin matrix simultaneously causes an increase of the volumetric contraction and of the elastic modulus [59]. In flowable composites, the reduced SS is attributable to the fact that the capacity of internal deformation is inversely proportional to the inorganic filler content [60]. Moreover, polymerization kinetics can influence the SS [61]. A previous study conducted by Ilie and Hickel [9] compared the SS and micromechanical properties of a bulk-fill flowable resin composite (SDR) to traditional flowable and non-flowable composites. SDR showed significantly lower polymerization stress, as observed in the present study, but lower micromechanical properties than hybrid composites. Within the flowable composites, SDR flow achieved the lowest Vickers hardness, the highest modulus of elasticity, and the highest creep, and showed a significantly lower elastic deformation. The low polymerization shrinkage for SDR flow resulted from the addition of the “polymerization modulator”, a chemical moiety in the resin backbone that increases flexibility and thus relaxes the polymerized network without harming DC. 

The results of the present study are also in accordance with El-Damanhoury are Platt [46], who evaluated the polymerization SS kinetics of five low-shrinkage, light-cured bulk-fill resin composites (SDR, Tetric Bulk Fill, Venus Bulk-Fill, X-tra Fill, and Filtek Bulk Fill). The real-time SS of the investigated composites was measured using a tensometer, which showed that Venus Bulk Fill and SDR had significantly lower stress values during irradiation. The findings of El-Damanhoury and Platt [46] and the present study are in accordance with the fact that SDR and Venus Bulk Fill, despite being the materials with the lowest filler concentration (between 38% and 44% by volume, respectively) and with a substantial volumetric shrinkage compared to other resin composite flow [62], are designed to greatly reduce shrinkage stress.

## 5. Conclusions

All tested materials showed a different decrease in MH values along the lateral surface. This MH decrease became significantly different from the top surface at different level of depth for each material. Indeed, only low-viscosity bulk-fill materials confirmed the possibility of layering 4 mm layers, while high-viscosity bulk-fill composites showed an initial decrease of MH after 2 mm of depth, suggesting a potential reduction of the mechanical properties and, thus, of the chewing force resistance when employed in load-bearing contact areas. Venus Bulk Fill and SDR showed inferior hardness, but a significantly reduced SS compared to the other tested materials. This suggests that these bulk-fill materials should be employed as an internal layer in contact with cavity walls but not on load-bearing surfaces. Further in vitro evaluations, employing methods that better simulate clinical conditions and the aging effect on bulk-fill composites, should be conducted, as well as in vivo studies, which are necessary to clinically assess the behavior of these materials over time.

## Figures and Tables

**Table 1:** Materials composition.

Composite	Manufacturer	Type	Resin Matrix	Filler	Filler w%; v%
Tetric Evoceram Bulk Fill	Ivoclar Vivadent, Schaan, Liechtenstein	Nano-hybrid, high viscosity	Bis-GMA, UDMA	Ba-Al-Si-glass, prepolymer filler (monomer, glass filler and ytterbium fluoride). Spherical mixed oxide	79–81 (including 17% prepolymers); 60–61
SureFil SDR	Dentsply De Trey, Konstanz, Germany	Low viscosity	Modified UDMA, TEGDMA, EBPDMA	Ba-Al-F-B-Si glass and St-Al-F-Si glass as fillers	68; 44
X-tra Base	VOCO, Cuxhaven, Germany	Hybrid, low viscosity	Bis-GMA, UDMA	2-3 μm Ba B Al Si glass filler	75; 60
SonicFill	Kerr Corp. California USA	Nano-hybrid, high viscosity	Bis-GMA, TEGDMA, EBPDMA	SiO_2_, glass, oxide	83.5; 66
Filtek Bulk Fill	3M ESPE, St Paul, MN, USA	Nano-hybrid, low viscosity	Bis-GMA, UDMA, Bis-EMA, Procrylat resins	Zirconia/silica, ytterbium trifluoride	64.5; 42.5
Venus Bulk Fill	Heraeus Kulzer, Hanau, Germany	Nano-hybrid, low viscosity	multifunctional methacrylate monomers (UDMA, EBPDMA)	Ba-Al-F silicate glass, YbF_3_, SiO_2_	65; 38

UDMA—urethane dimethacrylate, TEGDMA—triethylene glycol dimethacrylate, EBPDMA—ethoxylated Bis-GMA, Bis-GMA—bisphenol A-glycidyl methacrylate.

**Table 2:** Mean values and standard deviation of Vickers hardness (VHN) registered in different groups for bottom, lateral, and top surfaces. Different superscript letters indicate significant difference within column (*p* < 0.05).

	Material					
	Venus Bulk Fill	SDR	Filtek Bulk Fill	Xtra Base	SonicFil	Tetric Bulk Fill
Top	32.8 ± 3.4 ^a^	54.0 ± 6.6 ^a^	97.7 ± 4.9 ^a^	91.5 ± 4.8 ^a^	92.5 ± 4.8 ^a^	101.5 ± 4.8 ^a^
1 mm	32.1 ± 6.1 ^a^	46.2 ± 6.1 ^a^	95.3 ± 3.3 ^a^	91.5 ± 3.7 ^a^	93.5 ± 4.8 ^a^	100.6 ± 6.4 ^a^
2 mm	29.4 ± 6.1 ^a^	44.2 ± 6.2 ^b^	91.5 ± 4.5 ^a^	89.5 ± 4.6 ^a^	92.5 ± 5.5 ^a^	99.7 ± 6.5 ^a^
3 mm	28.9 ± 5.4 ^a^	36.9 ± 6.4 ^b^	84.9 ± 7.8 ^b^	82.3 ± 2.6 ^b^	88.8 ± 7.1 ^a^	94.1 ± 6 ^a^
4 mm	27.4 ± 5.1 ^a^	33.0 ± 5.5 ^b^	75.1 ± 8.5 ^b^	76.7 ± 2.6 ^b^	86.2 ± 6.4 ^b^	90.2 ± 6.7 ^b^
5 mm	26.1 ± 3.5 ^b^	31.7 ± 4.7 ^b^	45.9 ± 8.5 ^b^	59.4 ± 5.4 ^b^	60.1 ± 4.7 ^b^	60.9 ± 8.3 ^b^
6 mm	24.4 ± 4.9 ^b^	29.5 ± 5.1 ^b^	44.3 ± 7.5 ^b^	56.3 ± 3.7 ^b^	54.6 ± 4.2 ^b^	58.6 ± 9.1 ^b^
Bottom	19.1 ± 3.4 ^b^	30.4 ± 4.5 ^b^	36.9 ± 9 ^b^	53.0 ± 6.2 ^b^	48.4 ± 6.5 ^b^	49.7 ± 6.6 ^b^

**Table 3:** Mean microhardness (MH at 80% of top MH and depth at 80% of max MH for bulk-fill composites).

	Venus Bulk Fill	SDR	Filtek Bulk Fill	Xtra Base	SonicFil	Tetric Bulk Fill
80% Top (VHN)	26.2	43.1	78.1	74.0	74.0	81.2
80% Top (mm)	4.92	2.15	3.69	4.16	4.47	4.31

**Table 4:** Mean values and standard deviation (SD) for shrinkage stress (SS) and time to achieve maximum stress rate (t-Max) of the tested materials. Different superscript letters indicate significant difference within column (*p* < 0.05).

Material	Contraction Stress (MPa)	t-Max (sec)
Venus Bulk	0.60 ± 0.03 ^a^	87.23 ± 2.76
SDR	0.61 ± 0.05 ^a^	77.12 ± 2.56
Filtek Bulk	0.88 ± 0.04 ^b^	73.34 ± 2.36
X-tra Base	0.89 ± 0.05 ^b^	75.08 ± 2.78
Sonicfil	0.94 ± 0.05 ^b^	30.29 ± 2.02
Tetric Bulk	0.82 ± 0.07 ^b^	29.43 ± 1.93

## References

[1] Kwon Y., Ferracane J., Lee I.B. (2012). Effect of layering methods, composite type, and flowable liner on the polymerization shrinkage stress of light cured composites. Dent. Mater..

[2] van Dijken J.W. (2010). Durability of resin composite restorations in high C-factor cavities: A 12-year follow-up. J. Dent..

[3] Krämer N., Lohbauer U., García-Godoy F., Frankenberger R. (2008). Light curing of resin-based composites in the LED era. Am. J. Dent..

[4] Chen H.Y., Manhart J., Hickel R., Kunzelmann K.H. (2001). Polymerization contraction stress in light-cured packable composite resins. Dent. Mater..

[5] Braga R.R., Ballester R.Y., Ferracane J.L. (2005). Factors involved in the development of polymerization shrinkage stress in resin-composites: A systematic review. Dent. Mater..

[6] Kim M.E., Park S.H. (2011). Comparison of premolar cuspal deflection in bulk or in incremental composite restoration methods. Oper. Dent..

[7] van Dijken J.W., Pallesen U. (2014). A randomized 10-year prospective follow-up of Class II nanohybrid and conventional hybrid resin composite restorations. J. Adhes Dent..

[8] Czasch P., Ilie N. (2013). In vitro comparison of mechanical properties and degree of cure of bulk fill composites. Clin. Oral Investig..

[9] Ilie N., Rencz A., Hickel R. (2013). Investigations towards nano-hybrid resin-based composites. Clin. Oral Investig..

[10] van Dijken J.W., Pallesen U. (2016). Posterior bulk-filled resin composite restorations: A 5-year randomized controlled clinical study. J. Dent..

[11] Zorzin J., Maier E., Harre S., Fey T., Belli R., Lohbauer U., Petschelt A., Taschner M. (2015). Bulk-fill resin composites: Polymerization properties and extended light curing. Dent. Mater..

[12] Moorthy A., Hogg C.H., Dowling A.H., Grufferty B.F., Benetti A.R., Fleming G.J. (2012). Cuspal deflection and microleakage in premolar teeth restored with bulk-fill flowable resin-based composite base materials. J. Dent..

[13] Ilie N., Hickel R. (2011). Investigations on a methacrylate-based flowable composite based on the SDR™ technology. Dent. Mater..

[14] Ilie N., Bucuta S., Draenert M. (2013). Bulk-fill resin-based composites: An in vitro assessment of their mechanical performance. Oper. Dent..

[15] Jung J.H., Park S.H. (2017). Comparison of Polymerization Shrinkage, Physical Properties, and Marginal Adaptation of Flowable and Restorative Bulk Fill Resin-Based Composites. Oper. Dent..

[16] Furness A., Tadros M.Y., Looney S.W., Rueggeberg F.A. (2014). Effect of bulk/incremental fill on internal gap formation of bulk-fill composites. J. Dent..

[17] Scougall-Vilchis R.J., Hotta Y., Hotta M., Idono T., Yamamoto K. (2009). Examination of composite resins with electron microscopy, microhardness tester and energy dispersive X-ray microanalyzer. Dent. Mater. J..

[18] El-Safty S., Akhtar R., Silikas N., Watts D.C. (2012). Nanomechanical properties of dental resin-composites. Dent. Mater..

[19] Taylor D.F., Kalachandra S., Sankarapandian M., McGrath J.E. (1998). Relationship between filler and matrix resin characteristics and the properties of uncured composite pastes. Biomaterials.

[20] Obici A.C., Sinhoreti M.A., Correr Sobrinho L., de Goes M.F., Consani S. (2004). Evaluation of depth of cure and Knoop hardness in a dental composite photo-activated using different methods. Braz. Dent. J..

[21] de Araújo C.S., Schein M.T., Zanchi C.H., Rodrigues S.A., Demarco F.F. (2008). Composite resin microhardness: The influence of light curing method, composite shade, and depth of cure. J. Contemp. Dent. Pract..

[22] Bouschlicher M.R., Rueggeberg F.A., Wilson B.M. (2004). Correlation of bottom-to-top surface microhardness and conversion ratios for a variety of resin composite compositions. Oper. Dent..

[23] Watts D.C. (2005). Reaction kinetics and mechanics in photo-polymerised networks. Dent. Mater..

[24] Frassetto A., Navarra C.O., Marchesi G., Turco G., Di Lenarda R., Breschi L., Ferracane J.L., Cadenaro M. (2012). Kinetics of polymerization and contraction stress development in self-adhesive resin cements. Dent. Mater..

[25] Bucuta S., Ilie N. (2014). Light transmittance and micro-mechanical properties of bulk fill vs. conventional resin based composites. Clin. Oral Invest..

[26] Leprince J.G., Palin W.M., Vanacker J., Sabbagh J., Devaux J., Leloup G. (2014). Physico-mechanical characteristics of commercially available bulk-fill composites. J. Dent..

[27] Rodriguez A., Yaman P., Dennison J., Garcia D. (2017). Effect of Light-Curing Exposure Time, Shade, and Thickness on the Depth of Cure of Bulk Fill Composites. Oper. Dent..

[28] Par M., Gamulin O., Marovic D., Klaric E., Tarle Z. (2014). Effect of temperature on post-cure polymerization of bulk-fill composites. J. Dent..

[29] Alrahlah A., Silikas N., Watts D.C. (2014). Post-cure depth of cure of bulk fill dental resin-composites. Dent. Mater..

[30] Li J., Li H., Fok A.S., Watts D.C. (2009). Multiple correlations of material parameters of light-cured dental composites. Dent. Mater..

[31] Tsai P.C., Meyers I.A., Walsh L.J. (2004). Depth of cure and surface microhardness of composite resin cured with blue LED curing lights. Dent. Mater..

[32] Flury S., Hayoz S., Peutzfeldt A., Hüsler J., Lussi A. (2012). Depth of cure of resin composites: Is the ISO 4049 method suitable for bulk fill materials?. Dent. Mater..

[33] Leprince J.G., Leveque P., Nysten B., Gallez B., Devaux J., Leloup G. (2012). New insight into the "depth of cure" of dimethacrylate-based dental composites. Dent. Mater..

[34] Musanje L., Darvell B.W. (2006). Curing-Light Attenuation in Filled-Resin Restorative Materials. Dent. Mater..

[35] Leloup G., Holvoet P.E., Bebelman S., Devaux J. (2002). Raman Scattering Determination of the Depth of Cure of Light-Activated Composites: Influence of Different Clinically Relevant Parameters. J. Oral Rehabil..

[36] Davidson-Kaban S.S., Davidson C.L., Feilzer A.J., de Gee A.J., Erdilek N. (1997). The effect of curing light variations on bulk curing and wall-to-wall quality of two types and various shades of resin composites. Dent. Mater..

[37] Unterbrink G.L., Muessner R. (1995). Influence of Light Intensity on Two Restorative Systems. J. Dent..

[38] Shortall A.C. (2005). How light source and product shade influence cure depth for a contemporary composite. J. Oral Rehabil..

[39] Felix C.A., Price R.B., Andreou P. (2006). Effect of reduced exposure times on the microhardness of 10 resin composites cured by high-power LED and QTH curing lights. J. Can. Dent. Assoc..

[40] Ferracane J.L., Greener E.H. (1984). Fourier transform infrared analysis of degree of polymerization in unfilled resins--methods comparison. J. Dent. Res..

[41] Hadis M.A., Shortall A.C., Palin W.M. (2012). Specimen Aspect Ratio and Light Transmission in Photoactive Dental Resins. Dent. Mater..

[42] Yoon T.H., Lee Y.K., Lim B.S., Kim C.W. (2002). Degree of polymerization of resin composites by different light sources. J. Oral Rehabil..

[43] AlQahtani M.Q., Michaud P.L., Sullivan B., Labrie D., AlShaafi M.M., Price R.B. (2015). Effect of High Irradiance on Depth of Cure of a Conventional and a Bulk Fill Resin-based Composite. Oper. Dent..

[44] Ilie N., Stark K. (2014). Curing behaviour of high-viscosity bulk-fill composites. J. Dent..

[45] Kumar N., Fareed M.A., Zafar M.S., Ghani F., Khurshid Z. (2020). Influence of various specimen storage strategies on dental resin-based composite properties. Mater. Tech..

[46] El-Damanhoury H., Platt J. (2014). Polymerization shrinkage stress kinetics and related properties of bulk-fill resin composites. Oper Dent..

[47] Finan L., Palin W.M., Moskwa N., McGinley E.L., Fleming G. (2013). The Influence of Irradiation Potential on the Degree of Conversion and Mechanical Properties of Two Bulk-Fill Flowable RBC Base Materials. Dent. Mater..

[48] Ilie N., Keßler A., Durner J. (2013). Influence of various irradiation processes on the mechanical properties and polymerisation kinetics of bulk-fill resin based composites. J. Dent..

[49] Spinell T., Schedle A., Watts D.C. (2009). Polymerization shrinkage kinetics of dimethacrylate resin-cements. Dent. Mater..

[50] Burgess J., Cakir D. (2010). Comparative properties of low-shrinkage composite resins. Compend. Contin. Educ. Dent..

[51] Braem M., Finger W., Van Doren V.E., Lambrechts P., Vanherle G. (1989). Mechanical properties and filler fraction of dental composites. Dent. Mater..

[52] Gonçalves F., Kawano Y., Braga R.R. (2010). Contraction stress related to composite inorganic content. Dent. Mater..

[53] Baroudi K., Saleh A.M., Silikas N., Watts D.C. (2007). Shrinkage behaviour of flowable resin-composites related to conversion and filler-fraction. J. Dent..

[54] Satterthwaite J.D., Maisuria A., Vogel K., Watts D.C. (2012). Effect of resin-composite filler particle size and shape on shrinkage-stress. Dent. Mater..

[55] Condon J.R., Ferracane J.L. (2000). Assessing the effect of composite formulation on polymerization stress. J. Am. Dent. Assoc..

[56] Kleverlaan C.J., Feilzer A.J. (2005). Polymerization shrinkage and contraction stress of dental resin composites. Dent. Mater..

[57] Marchesi G., Breschi L., Antoniolli F., Di Lenarda R., Ferracane J., Cadenaro M. (2010). Contraction stress of low-shrinkage composite materials assessed with different testing systems. Dent. Mater..

[58] Haak R., Wicht M.J., Noack M.J. (2003). Marginal and internal adaptation of extended class I restorations lined with flowable composites. J. Dent..

[59] Braem M., Lambrechts P., Vanherle G., Davidson C.L. (1987). Stiffness increase during the setting of dental composite resins. J. Dent. Res..

[60] Vaidyanathan J., Vaidyanathan T.K. (2001). Flexural creep deformation and recovery in dental composites. J. Dent..

[61] Lim B.S., Ferracane J.L., Sakaguchi R.L., Condon J.R. (2002). Reduction of polymerization contraction stress for dental composites by two-step light-activation. Dent. Mater..

[62] Michaud P.L., Price R.B., Labrie D., Rueggeberg F.A., Sullivan B. (2014). Localised irradiance distribution found in dental light curing units. J. Dent..

